# Treatment of large, chronic and persistent macular hole with internal limiting membrane transposition and tuck technique

**DOI:** 10.1186/s40942-019-0206-7

**Published:** 2020-03-09

**Authors:** Nicholas S. K. Fung, Anthony K. H. Mak, Rachel Yiu, Ian Y. H. Wong, Wai Ching Lam

**Affiliations:** 1 The University of Hong Kong, Hong Kong, SAR China; 2 Grantham Hospital, Hong Kong, SAR China

## Abstract

**Background:**

Large, chronic full thickness macular holes which failed previous treatments are difficult to manage and even left untreated due to poor prognosis. A retrospective review of consecutive cases with chronic (at least 1 year) full thickness macular holes and internal limiting membrane (ILM) free flap transposition with tuck technique, after previously failed vitrectomy.

**Methods:**

This was a retrospective and interventional study conducted in a single centre by a single surgeon. Patients with full thickness macular hole for at least 1 year and at least one previously failed vitrectomy with ILM peeling were recruited. A 25G vitrectomy with ILM free flap transposition was done without assistance of PFCL, viscoelastic or autologous blood. The free flap was manually tucked into the macular hole free space and gas fluid exchange was performed with 20% SF6 as tamponade. The patients were postured prone for 2 weeks postoperatively. Best corrected visual acuity, macular hole duration, previous surgeries, optical coherence tomography (OCT) appearance, hole size and closure rate were recorded.

**Results:**

8 consecutive patients were included from May 2016 to Feb 2018. Transposition surgery was performed an average of 1481 days (SD 1096) after diagnosis of macular hole and average of 1226 days (SD 1242) after first vitrectomy. Macular hole mean size was 821 μm (SD 361.3), preoperative VA was logMAR 1.038 (SD 0.19), postoperative VA was logMAR 0.69 (SD 0.19) at 3 months. There were 1.13 lines gained and a significant improvement of logMAR 0.33 (p = 0.0084) at 6 months. Hole closure was seen in 7 out of 8 eyes (87.5%). The OCT with failed closure showed ILM flap within a flat hole, however no overlying neurosensory layers was seen. The duration from diagnosis to surgery was 2349 days in this case.

**Conclusion:**

Free flap ILM transposition tuck without the use of additional intraoperative tamponade is an effective technique in treating large chronic macular holes with previously failed primary macular hole surgeries.

*Trial registration* (IRB of the Hong Kong University and Hospital Authority Hong Kong West Cluster, ref UW19-440), June 17, 2019.

## Background

Since its introduction in the 1990’s [1], pars plana vitrectomy, with internal limiting membrane (ILM) peeling and gas tamponade has been widely regarded as the gold standard procedure for surgical treatment of macular hole. Despite high rates of success, the reported rate of persistent macular hole after primary surgery varies between 8 and 44% [12]. In particular, large macular holes, myopic macular holes and retinal detachments associated with macular holes were all associated with poorer visual outcome and lower macular hole closure rates. This has led to modification of conventional macular hole surgery, such as inverted ILM flap, to improve success. Some macular holes, however, are still persistent after primary vitrectomy with ILM peeling; treatment for these cases remain a surgical challenge. Due to lack of randomized control trials and small sample sizes of existing case series, there is currently no consensus on the best technique for the treatment of persistent macular holes.

The inverted ILM flap was first described by Michalewska et al. [2] in 2010 showed promising results for large macular holes. The closure rate after inverted ILM flap procedure was reported to be 98% when the procedure was successfully executed, compared to 88% observed in the control group which underwent conventional pars plans vitrectomy with ILM peeling and air injection. More importantly, inverted ILM flap resulted in fewer flat-open macular holes and better post-operative visual acuity. It is postulated that the ILM flap serves as a scaffold for glial cell proliferation, allowing Müller cells and photoreceptors to assume anatomical positions more akin to healthy foveola. This theory is supported by clinical observation of faster recovery time with macular holes covered by ILM flaps [3], and vice versa [4]. The conception of the inverted ILM flap technique paved way for further modification. Apart from large macular holes, modified ILM flap techniques have been used to treat macular holes in high myopes with sound improvement in outcomes [5, 6]. Some of the modifications include using a larger flap [5], using autologous blood to reduce the chance of ILM flap displacement [6, 12], and temporal inverted ILM flap to minimize iatrogenic trauma induced by ILM peeling [13].

More recently, the advent of autologous transplantation of free ILM flap has provided newfound optimism in improving visual and anatomical outcomes for persistent macular holes after primary surgery [7, 8, 14, 15]. With this development, however, came a brand-new set of surgical challenges and direction for further research [9]. In the past, it has been suggested that ILM should be peeled as far to the arcade as possible, to relieve tangential traction, thereby improving outcome of macular hole surgery [10]. As a result of this contentious theory, some patients are left with limited residual ILM after primary surgery; therefore, harvesting a free ILM flap can be challenging. Furthermore, securing the free ILM flap in place is more difficult compared to the conventional inverted ILM flap technique. Three different prospective interventional case series have reported the use of viscoelastic to secure free ILM flaps into macular holes. The successful hole closure rate was reported to be between 90 and 92% [11–15]. The surgical techniques employed were similar in all 3 case series, 2 of them used viscoelastic to secure the free flap while 1 used the tuck technique. The use of perfluoro-*n*-octane has also been described in a case reported to aid the anchoring of the free ILM flap, with successful hole closure and improvement in visual acuity [16]. De Novelli et al. have described 100% macular hole closure rate in a case series, using autologous ILM transplantation to treat large, chronic or persistent macular holes, without the aid of viscoelastic [15]. Four of the ten cases in that series were treated for recurrent or persistent macular holes with improvement in visual acuity. In this study, we describe a similar method of autologous ILM transplantation without the use of viscoelastic, specifically for the treatment of persistent, large, chronic full-thickness macular holes.

## Methods

This was a retrospective and interventional study conducted in a single centre by a single surgeon. The study protocol followed the principles of the Declaration of Helsinki and was approved by the institutional research ethics board (IRB of the Hong Kong University and Hospital Authority Hong Kong West Cluster, ref UW19-440).

Consecutive patients from May 2016 to February 2018 with persistent full thickness macular hole for at least 1 year and at least one previous vitrectomy with ILM peeling were recruited. A 25 Gauge vitrectomy (Alcon Constellation^®^ vision system) with ILM free flap transposition was performed without secondary assistance of PFCL, viscoelastic or autologous blood. The optimal free flap diameter was 1.5 times the size of the macular hole and was harvested outside the arcades or temporal to the macula with an Alcon Grieshaber Revolution^®^ DSP ILM forceps or DORC Disposable Microforceps: ILM 25G/0.5 mm. The ILM leading edge of the free flap was manually tucked into the macular hole followed by 360 tucking of the flap with a closed ILM forceps tip. Finally gas fluid exchange was performed using a soft tip back flush and 20% SF6 was used as tamponade. The patients were postured prone for 2 weeks postoperatively. None of the surgeries involved additional corneal procedures or cataract removal since all cases were pseudophakic.

All patients had a complete ophthalmic evaluation at before surgery, and at 3 and 6 months after surgery including best corrected visual acuity (BCVA), macular hole duration, previous ophthalmic surgeries, Optical Coherence Tomography (SD–OCT) appearance hole size and closure rate were recorded. We recorded Snellen BCVA, which was converted to LogMAR for statistical calculations. As all cases were vitrectomized with full thickness macular holes, it was more relevant to use the OCT appearance [17] to classify the FTMH rather than the OCT based classification proposed by the International Vitreomacular Traction Study (IVTS) group [18].

- Type 1—macular holes with cystic edema of the neurosensory retina on both margins of the hole on both the horizontal and vertical scans.
- Type 2—macular holes with cystic edema of the neurosensory retina on only one margin of the hole on either the horizontal or vertical scan.
- Type 3—macular hole with full-thickness defect of neurosensory retina without cystic edema or detachment of the margins.
- Type 4—macular hole with localized detachment of the neurosensory retina at the margin without cystic edema.
- Type 5—macular hole with thinning of the neurosensory retina.

Statistical analysis including Student’s T test was used to compare visual acuity before and after surgery. Pearson’s and Spearman’s correlation were calculated on GraphPad Prism v6 (GraphPad Software, San Diego, CA), for continuous and discrete data respectively. Continuous data were expressed as mean and discrete data were expressed as percentages. A p value of < 0.05 was considered to be statistically significant.

## Results

Eight consecutive patients were included from May 2016 to Feb 2018. 50% right eye, 71% female with an average age of 66 years old (± 7). Transposition surgery was performed at an average of 1481 days (SD 1096) after diagnosis of macular hole and at an average of 1226 days (SD 1242) after first vitrectomy.

Macular hole average size was 821 μm (SD 361.3), preoperative VA was logMAR 1.038 (SD 0.19), postoperative VA was logMAR 0.69 (SD 0.19) at 3 months. There were 1.13 lines gained with a significant improvement of logMAR 0.33 (p = 0.0084) at 6 months. Hole closure was seen in 7 out of 8 eyes (87.5%) (Table 1).

**Table 1:** Macula hole features and results

Patient ID	Age	Duration of macula hole	Duration since PPV	Macula hole size (μm)	Pre op BCVA	Post op BCVA	VA gain (lines)	Hole closure	Ocular co morbidity
1	61	399 days	372 days	440	1.0	0.48	2	Y	Macula off retinal detachment
2	69	1013 days	722 days	830	1.3	0.7	1.5	Y	Nil
3	65	2349 days	2330 days	1210	1.0	0.7	1	N	High myopia
4	56	Since 2015	Unknown	1370	1.0	0.48	2	Y	Entropion with keratopathy
5	72	736 days	471 days	340	1.0	0.48	2	Y	Advanced glaucoma
6	68	993 days	210 days	747	1.3	1.0	1	Y	High myope and amblyopia
7	73	1333 days	938 days	617	0.7	0.7	0	Y	
8	76	3542 days	3542 days	1014	1.0	1.0	0	Y	High myope
Average	66.3	1481 days	1226 days	821	1.04	0.69	1.19	87%	

Macular holes that were more chronic were correlated with larger holes (0.774, p = 0.0410) and fewer lines gained (− 0.774, p = 0.0411). It was also noted that longer chronicity was correlated to poorer macular hole OCT classification, i.e. Type 4 and 5 (0.896, p = 0.019) (Table 2). There were no significant correlations between closure rate and macular hole duration or hole size (− 0.435, p = 0.281).

**Table 2:** Duration Pearson’s correlation, except ^a^ Spearman

	Correlation	p value
Hole size	0.774	0.041*
Pre op BCVA	− 0.178	0.703
Post op BCVA	0.623	0.135
VA gain (lines)	− 0.774	0.041*
Hole closure	− 0.349	0.442
Hole type^a^	0.896	0.019*

*Statistically significant

**Fig. 1:**
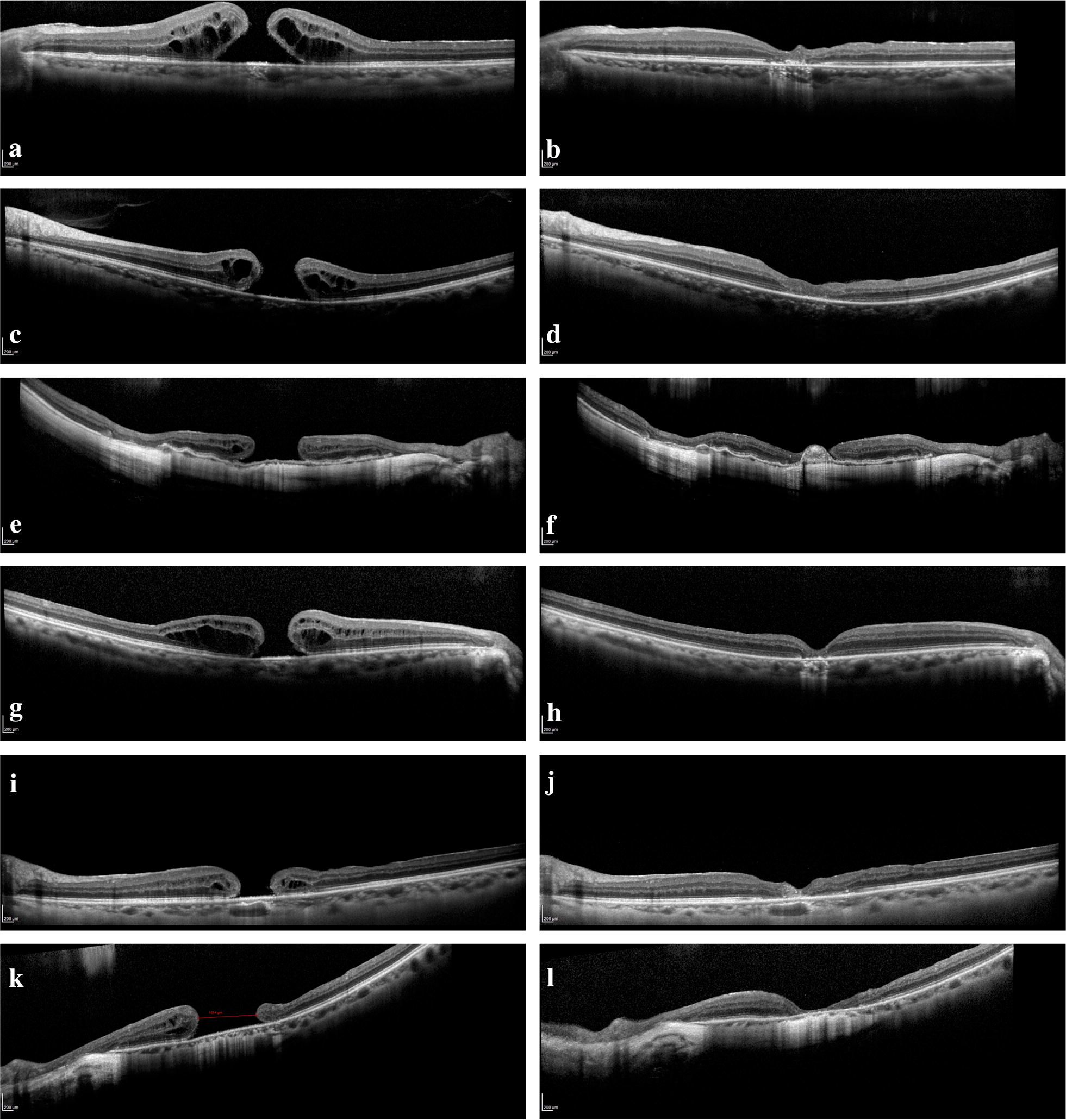
**a**–**d** Patient 1 and 2 with pre op OCT showing type 1 holes and post op OCT showing a closed macular hole. **e**, **f** Patient 3 with pre op OCT showing a type 3 hole and post op OCT showing ILM material but no neuroretinal tissue overlying the smaller hole (failed closure). **g**, **h** Patient 6 with pre op OCT showing a type 1 hole and post op OCT showing a closed macular hole. **i**, **j** Patient 7 with pre op OCT showing a chronic (1333 days) type 3 hole (617 μm) with closure seen on the post op OCT. **k**, **l** Patient 8 with pre op OCT showing a large (1014 μm), chronic (1481 days) type 3 hole with closure on the post op OCT showing a thin layer of overlying continuous neuroretinal layer 209 × 296 mm (300 × 300 DPI)

The eye with failed closure showed ILM flap material within a flat hole on OCT, however no overlying neurosensory retinal layers were seen. The duration from diagnosis to surgery was 2349 days in this case (Fig. 1 e, f).

## Discussion

Primary treatment for macular hole has been well established even for large chronic holes. However, an effective treatment for persistent large chronic holes which have previously failed vitrectomy and ILM peeling has not been standardized. This study shows that successful closure with visual acuity improvement can be achieved even in patients with very large, chronic and previously treated macular holes.

Patients 1 and 5 had relatively smaller holes (440 μm and 340 μm respectively) and showed the most improvement in visual acuity, however, correlations between hole size and BCVA improvement was not significant (− 0.22, p = 0.597) (Fig. 1a, b). Patients 2 (Fig. 1c, d) and 6 (Fig. 1g, h) have relatively larger holes (830 μm and 747 μm respectively) and 3 years of chronicity, but still showed closure and BCVA improvement. Patient 6 in particular was also suffering from extreme myopia with an axial length of 33.1 mm, which made it difficult to harvest a usable ILM flap and reach the hole with standard forceps. However, it is evident that chronicity significantly affects the BCVA improvement as seen in patients 3, 7 and 8 (Pearson’s = − 0.774) (Fig. 1e, f, i–l). It is interesting to see that despite non-closure of the macular hole in patient 3, the improvement in hole size and configuration resulted in improved BCVA.

The timing of vitrectomy for patient 4 is unknown since she was not able to recall the date and the procedure was performed at another institution. The duration was not included in the calculation for chronicity although we know that the hole was present for at least 2 years since we had her OCT scan from 2015.

Amongst our patients included, all but one of the primary vitrectomies were performed by other surgeons. Therefore, the initial peel and exact techniques used were unknown. The duration of the macular hole was also longer than the stated time, as the date of diagnosis in our clinic was used, which was delayed after a referral waiting period. However, no additional corneal surgery, lens exchange or ILM peeling were done in addition to the harvesting of the graft. Any improvement in BCVA and hole closure would not be affected by secondary maneuvers.

Although a free flap ILM transposition in theory will help to close previously peeled and vitrectomized eyes, there are several potential challenges associated with this technique. One difficulty in this surgery lies in harvesting a viable ILM graft in patients with the appropriate size. The author prefers to use 1.5 times the size of macular hole to reduce redundancy in overly large flaps or residual space in small grafts as was the situation in patient 8. Secondly, tucking of a curled, soft and pliable ILM into a macular hole can be challenging and requires some patience. The author finds that tucking the leading edge with the forceps open will allow the ILM to adhere to the hole cavity, and dislodge more easily from the forceps. A 25G forceps with rounder edges and lower clamping power will reduce the chance of the flap adhering to the forceps, while providing a smoother surface to tuck the flap edges. The ILM flap often remains adherent even after the opening of the forceps. This can usually be resolved by using the light pipe to gently disengage the flap. When tucking the flap up against the wall of the macular hole, care needs to be taken to avoid applying direct pressure onto the RPE below, which can cause damage to the layer. This maneuver, similar to macular hole tapping, may also cause the macular hole wall to sit higher and increase the Macular hole index (MHI), thereby increasing the chance of closure as shown by Kusuhara et al. [19] Finally, to reduce turbulence and dislocation of the transposed ILM flap, gas fluid exchange should be performed at the surface of the fluid level, with passive aspiration towards the end, and scleral indentation should be avoided after ILM flap transposition.

Despite being shown to be an effective surgery to treat chronic, large macular holes with failed primary surgery in this study, ILM transposition has not been proven to be an effective adjunct technique in primary surgeries such as the inverted ILM flap and its variations. For non-chronic macular holes, the technique of tucking ILM flap in the macular hole has been controversial [20]. It has been shown that ILM tucking as described by the Michalewska was not essential, and inverted flap without manipulation produced similar macular hole closure rate [21]. However, for chronic or recurrent macular holes, it would be difficult to conduct a similar randomized control trial, because of the low sample size.

For persistent macular who failed primary surgery, other options include autologous blood plug, and neurosensory retinal flap. Grewal and Mahmoud [16] described the technique of autologous retinal transplant to treat refractory macular holes, with promising outcome. Anatomical closure was achieved in 87.8% of the 41 patients in this retrospective study, which was very similar to the closure rate of 87.5% we observed in our study. The visual acuity was improved in 36.6% of patients, and some 13.8% were reported to have decreased in vision despite successful anatomical outcome. Furthermore, harvesting the retinal graft is not without its risks. Retinal detachment, vitreous haemorrhage and cystoid macular oedema were some of the complications reported in that study. The surgical methods described in our study can potentially be an alternative with slightly lower rates of complication and better visual outcomes.

Due to the chronicity of the disease, there was limited ellipsoid layer recovery seen on OCT even at 6 months post-operation. This is likely to have affected the visual acuity improvement. More data will be needed to understand the benefits this surgery including the closure mechanism and behavior of autologous ILM scaffolding.

## Conclusion

The majority of primary macular hole surgery is often straightforward for small, non-chronic cases. This study shows that the need for additional maneuvers such as inverted flaps may not be necessary even in chronic and large holes, when ILM transposition and tucking is an effective rescue surgery. Even greater success with this technique has been seen in non-chronic cases, as shown by Pires et al. [14] and De Novelli et al. [15], with anatomical closure rate of 91% and 100% respectively, and significant BCVA improvements in both studies. Although there is a steep learning curve, this technique is shown to be effective in challenging cases and beneficial in all spectrums of secondary repairs of macular holes.

## Data Availability

The datasets used and/or analysed during the current study are available from the corresponding author on reasonable request.
